# Gaps in the implementation and uptake of maternal nutrition interventions in antenatal care services in Bangladesh, Burkina Faso, Ethiopia and India

**DOI:** 10.1111/mcn.13293

**Published:** 2021-11-23

**Authors:** Tina Sanghvi, Phuong H. Nguyen, Manisha Tharaney, Sebanti Ghosh, Jessica Escobar‐Alegria, Zeba Mahmud, Tamirrat Walissa, Maurice Zafimanjaka, Sunny Kim

**Affiliations:** ^1^ Alive & Thrive Initiative FHI Solutions Washington District of Columbia USA; ^2^ Poverty, Health and Nutrition Division International Food Policy Research Institute Washington District of Columbia USA

**Keywords:** antenatal care (ANC), breastfeeding, dietary counselling, implementation, maternal nutrition, micronutrients, pregnancy weight gain

## Abstract

Antenatal care (ANC) is the largest health platform globally for delivering maternal nutrition interventions (MNIs) to pregnant women. Yet, large missed opportunities remain in nutrition service delivery. This paper examines how well evidence‐based MNIs were incorporated in national policies and programs in Bangladesh, Burkina Faso, Ethiopia and India. We compared the nutrition content of ANC protocols against global recommendations. We used survey data to elucidate the coverage of micronutrient supplementation, weight gain monitoring, dietary and breastfeeding counselling. We reviewed literature, formative research and program assessments to identify barriers and enabling factors of service provision and maternal nutrition practices. Nutrition information in national policies and protocols was often fragmented, incomplete and did not consistently follow global recommendations. Nationally representative data on MNIs in ANC was inadequate, except for iron and folic acid supplementation. Coverage data from subnational surveys showed similar patterns of strengths and weaknesses. MNI coverage was consistently lower than ANC coverage with the lowest coverage of weight gain monitoring and variable coverage of dietary and breastfeeding counselling. Key common factors associated with coverage were micronutrient supply disruptions; suboptimal counselling on maternal diet, weight gain, and breastfeeding; and limited or no record keeping. Adherence of women to micronutrient supplementation and dietary recommendations was low and associated with late and too few ANC contacts, poor maternal knowledge and self‐efficacy, and insufficient family and community support. Models of comprehensive nutrition protocols and health systems that deliver maternal nutrition services in ANC are urgently needed along with national data systems to track progress.

## Key points

- Delivering nutrition services to all pregnant women is essential for maternal and child health outcomes but remains a challenge.
- Nutrition gaps in antenatal care (ANC) include lack of specificity in national guidelines and protocols, bottlenecks in micronutrient supplies, low ANC provider knowledge and skills, inadequate supervision to reinforce counseling, and not engaging families to encourage key practices.
- National protocols for ANC should be more specific for the four nutrition interventions (micronutrient supplements, weight gain monitoring, counseling on diets and counseling on breastfeeding) and assign accountability for coverage and quality.
- Country models are needed for improving provision and utilisation of nutrition interventions through ANC that are based on comprehensive policy frameworks.

## 1 INTRODUCTION

Maternal nutrition is associated with women's health and well‐being, newborn survival, and child growth and development (Black et al., 2013). Despite improvements in our understanding of the problem, poor nutrition among women remains a significant global concern (Victora et al., 2021). The prevalence of low body mass index (BMI) among women has declined globally, but is still high in many low‐ and middle‐income countries (LMICs), particularly in South Asia (at 24%) and parts of sub‐Saharan Africa (at 20%) (Victora et al., 2021). The prevalence of anaemia is unacceptably high, affecting 30% of women of reproductive age (equivalent to over half a billion women aged 15–49 years), and 36.5% of pregnant women (WHO, 2021). Presence of both undernutrition and overweight and obesity in women is becoming more prevalent in some countries in Asia and sub‐Saharan Africa (Popkin et al., 2020).

Evidence reported in the Lancet nutrition series in 2008 (Bhutta et al., 2008), 2013 (Bhutta et al., 2013) and reiterated in 2021 (Keats et al., 2021) suggests that a package of essential nutrition interventions including micronutrient supplementation and nutrition counselling, delivered with high quality through antenatal care (ANC), can contribute to improving maternal nutrition. Findings from 81 LMICs estimate that if health systems could effectively deliver a subset of evidence‐based interventions to mothers and their newborns who are already seeking care, there would be a 28% decline in maternal and neonatal deaths, and 22% decrease in stillbirths (Chou et al., 2019). Despite this evidence, the coverage of nutrition interventions is far lower than the reach of their associated health service platforms (Heidkamp et al., 2020). In India (UP state), only half of pregnant women who attended ANC reported consuming iron and folic acid (IFA), and substantially fewer women initiated breastfeeding within one hour after delivery, as compared with the proportion of women who delivered in health facilities (Nguyen et al., 2021). Reducing missed opportunities for nutrition in health services offers high potential payoffs for improving maternal and infant nutrition (Heidkamp et al., 2021).

Understanding why, where and when the gaps occur in delivering nutrition interventions through ANC is an important next step for reducing missed opportunities. Before the publication of the WHO ANC guidelines in 2016, nutrition interventions received little priority in ANC services. Thus, there is scarce evidence on the content, quality, and factors associated with maternal nutrition interventions (MNIs) during ANC (Benova et al., 2018; Torlesse et al., 2021), other than IFA supplementation (Kavle & Landry, 2018; Sanghvi et al., 2010; Sununtnasuk et al., 2016). This paper provides details on the package of four MNIs as part of ANC in two sub‐Saharan and two South Asian countries (Bangladesh, Burkina Faso, Ethiopia and India). Specifically, we (1) elucidate the content of national policies and protocols on micronutrient supplementation, weight gain monitoring, and counselling on maternal diet and breastfeeding, (2) examine service delivery gaps in MNIs during ANC, and (3) identify factors associated with delivery of MNIs and maternal nutrition practices.

## 2 METHODS

We used mixed methods for the study. First, we conducted a desk review of maternal nutrition program and policy documents including ANC guidelines and protocols to identify nutrition contents in each country and compared these contents with the WHO ANC recommendations (WHO, 2016). Then we conducted secondary data analysis of national and subnational surveys to determine coverage of services delivered by ANC providers and nutrition practices among pregnant women. Finally, we reviewed data from multiple sources (peer‐reviewed publications, formative research reports and notes of stakeholder dialogues) to identify barriers and enabling factors that helped to explain the nutrition service delivery and maternal nutrition practices.

Four countries (Bangladesh, Burkina Faso, Ethiopia and India) were selected based on where the Alive & Thrive initiative had conducted or is conducting implementation research studies on maternal nutrition. These countries reflected poor maternal health and nutrition indicators (WHO UNICEF UNFPA World Bank Group & United Nations Population Division, 2019), high or rising proportion of ANC utilisation (EPHI & ICF, 2019; IIPS, 2017; INDS & ICF, 2012; NIPORT, 2020), and availability of recent information on the four priority MNIs (Kim, Ouédraogo, et al., 2020; Kim, Sununtnasuk, et al., 2020; Nguyen et al., 2015, 2018) (Table S1). The interventions were based on the WHO ANC guidelines (WHO, 2016) and endorsed by the Ministries of Health, including micronutrient supplementation, weight gain monitoring, dietary counselling, and counselling on breastfeeding.

Based on the WHO building blocks for health systems strengthening (WHO, 2010), we expected that maternal nutrition policies and protocols combined with the availability of supplies, skilled providers, supervision, monitoring, and utilisation of ANC services would result in a high level of service delivery and utilisation of nutrition interventions by pregnant women. Building on our previous work on infant and young child feeding interventions (Menon, Nguyen, Saha, Khaled, Kennedy, et al., 2016; Menon, Nguyen, Saha, Khaled, Sanghvi, et al., 2016; Sanghvi et al., 2016), we expected that maternal nutrition practices would likely be influenced by individual and household factors including knowledge, self‐efficacy, beliefs, access, and family support. Additionally, important community‐level factors might influence the extent to which pregnant women adopt recommended practices include food taboos or misconceptions on diets (Lakshmi, 2013; Zerfu et al., 2016) and strong gendered social norms with low priority given to women's nutrition (ROSHNI, 2019).

### 2.1 Policies and protocols

The Maternal Health and Nutrition Departments of the Ministries of Health provided the latest government policies, guidelines, protocols and directives for nutrition interventions in ANC. We consulted staff from the Ministries of Health to clarify terminology, overlapping or conflicting or missing content in policies and protocols. For information on the modes of service delivery for each intervention, we consulted government staff and other stakeholders working on ANC at the central and district levels.

We used a detailed checklist (Table 1) to compare the content of policies and protocols to global recommendations. For micronutrient supplementation, we reviewed the guidelines related to the types of micronutrients (IFA and/or calcium), the dose, the frequency of taking supplements (once or twice daily), and duration of supplementation. For weight gain monitoring and counselling, we reviewed guidelines on weight gain calculation and counselling. For dietary counselling, we reviewed content related to quantity and diversity of the diet during pregnancy. Lastly, we reviewed the counselling guidelines for preparing pregnant women for initiation of breastfeeding after birth and exclusive breastfeeding. For each service listed above, we also documented the modes of service delivery including types of service providers, locations of services (health facility or health service outreach sites in the community or home visits), timing and frequency of services, and ways to organise services (interpersonal or group, or community events). The information was organised by intervention for each country.

**Table 1:** Checklist for reviewing the protocols and programs for maternal nutrition interventions in ANC services

Interventions	Content of protocols and programs
*Micronutrient supplementation*	
Protocols	Supplement specifications, for example, 60 mg or 30–60 mg IFA and 400 mcg folic acid
	Total number of doses per pregnancy and daily/weekly frequency
	Counselling on adherence
Service delivery guidelines	Provision at no cost or fee charged for supplements
	Number of tablets given per ANC contact/visit
	Lay workers allowed to distribute supplements or not
	Facility‐based distribution only or community level distribution allowed
	Counselling quality specified, messages include managing side effects, risks of non‐adherence, how/when to take the tablets, total tablets in pregnancy. Interactive dialogue with Q&A
	Record keeping and use of data specified, including sources, interpretation, data reviews and actionable outcomes
Monitoring indicators	Consumption of any IFA/micronutrient tablets in the last pregnancy
	Consumption of 90 or more IFA/micronutrient tablets in the last pregnancy
	Counselling on IFA/micronutrients provided/received during ANC
*Weight gain monitoring*	
Protocols	Range of healthy weight gain (total and monthly) specified
	Weight gain tailored to individual pre‐ or early pregnancy BMI
	Method of calculating weight gain specified
	Referral criteria for excessive or low weight gain
	Counselling for maintaining healthy weight gain
Service delivery guidelines	Sites for weight taking are specified: facility, outreach, group sessions, home visits, and type of health worker
	Counselling quality specified, messages include woman's weight gain, healthy weight gain, diet and physical activity recommendations. Interactive dialogue with Q&A
	Record keeping and use of data specified, including sources, interpretation, data reviews and actionable outcomes
Monitoring indicators	Weight taken during ANC
	Number of times weight was taken and weight gain recorded
	Counselling provided/received on healthy weight gain
*Dietary counselling*	
Protocols	Counselling on the importance of consuming one food daily from five different healthy food groups, or names of local foods from different food groups
	Counselling on the importance of increasing the number of meals (and snacks) and the amount of food per meal by trimester to stay within a healthy range for weight gain
Service delivery guidelines	Site(s) and health workers for dietary counselling are specified: facility, outreach, group sessions, home visits
	Record keeping and use of data specified, including sources, interpretation, reviews and actionable outcomes
	Counselling quality specified, messages include food amounts/meals and snacks, and dietary diversity. Interactive dialogue with Q&A
Monitoring indicators	Counselling received on dietary diversity
	Counselling received on number of meals/snacks and amounts
*Breastfeeding counselling*	
Protocols	Counselling on the importance of early initiation within 1 h and how to place the newborn on the chest with the support of a health provider, preventing and managing common difficulties
	Counselling on the importance of six months of exclusive breastfeeding, skills (position, attachment, manual expression) and how to address common breastfeeding difficulties
Service delivery guidelines	Site(s) and health workers for breastfeeding counselling are specified: facility, outreach, group sessions, home visits
	Counselling quality specified, messages include early initiation and exclusive breastfeeding for 6 months. Interactive dialogue with Q&A
	Record keeping and use of data specified, including sources, interpretation, reviews and actionable outcomes
Monitoring indicators	Counselling received on early initiation of breastfeeding
	Counselling received on exclusive breastfeeding for 6 months

### 2.2 Nutrition services delivered in ANC and maternal nutrition practices

Data on the delivery of maternal nutrition services during ANC was compiled from the most recent nationally representative surveys including three Demographic and Health Surveys in Bangladesh (NIPORT, 2020), Ethiopia (EPHI & ICF, 2019), India (IIPS, 2017), and one national nutrition survey in Burkina Faso (PMA2020, 2018). Additional information was available from subnational surveys conducted by the International Food Policy Research Institute (IFPRI) as baselines for Alive & Thrive's MNI evaluations in the four countries (Kim, Ouédraogo, et al., 2020; Kim, Sununtnasuk, et al., 2020; Nguyen et al., 2015, 2018). These surveys used standardised methods and were comparable across countries.

Indicators for coverage of ANC and nutrition service delivery used self‐reported data based on interviews with women who received services during pregnancy for their last child in the past five years (national surveys) or in the past 6 months (subnational surveys). We first extracted data on the following indicators: percentage of women who attended any ANC from a skilled provider, completed four or more ANC visits, and started ANC in the first trimester of pregnancy. We then compiled information related to MNIs including percentage of women who consumed any IFA and received counselling on: IFA supplements, weight gain, diet, and breastfeeding.

### 2.3 Factors associated with nutrition service delivery and maternal nutrition practices

We searched PubMed and Global Health databases to identify articles published between January 2010 and April 2021. Search terms were applied for nutrition in ANC, micronutrient supplementation, weight gain monitoring, dietary counselling, and counselling on breastfeeding. In addition, grey literature published between January 2010 and April 2021, survey reports, and formative research studies were sourced from the Alive and Thrive website and document archives. Eligibility criteria included country (Bangladesh, Burkina Faso, Ethiopia, or India), year completed or published (2010–2021), content related to factors associated with either nutrition service delivery or maternal nutrition practices related to at least one of the four interventions. The information was organised by author, location, study design and approach, intervention(s) addressed, and key findings.

### 2.4 Ethical considerations

Ethical approval was not required for this study as anonymized data used for secondary data analysis were from existing national and subnational surveys, which all had prior ethical clearance from international and national institutional review boards.

## 3 RESULTS

### 3.1 National policies and guidelines on MNIs

Information in national protocols and policies on key nutrition interventions was often not available in a consolidated form but fragmented and scattered across several documents (Table 2, footnotes). The level of detail on nutrition related policies and protocols varied across interventions and countries. National protocols deviated from global recommendations in some countries due to the lack of clarity about global guidelines, shortage of resources including insufficient and overburdened service providers, and/or perceived low relevance.

**Table 2:** Content of national policy documents and service delivery practices related to maternal nutrition interventions in Bangladesh, Burkina Faso, Ethiopia and India

	Bangladesh	Burkina Faso	Ethiopia	India
*Micronutrient supplementation*				
Protocol: type, dose, frequency and duration	‐Daily IFA tablet with 60 mg iron and 400 mcg folic acid, from the day the pregnancy is identified	Daily IFA tablet with 60 mg iron and 400 mcg folic acid, starting from the first ANC visit until 42 days after delivery	Daily IFA tablet with 30–60 mg of iron and 400 mcg folic acid; complete at least 90 days of supplementation	‐Daily IFA tablet with 60 mg iron and 400 mcg folic acid, starting from the 2nd trimester for a total of 180 IFA tablets
	‐Two daily calcium tablets, each tablet containing 750 mg to 1 g elemental calcium, after the 1st trimester for a total of 360 tablets			‐Two daily calcium tablets, each tablet containing 500 mg elemental calcium and 250 IU vitamin D3, starting from the 2nd trimester for a total of 360 tablets
Service delivery	ANC providers should distribute a monthly supply of 30 IFA and 60 calcium, free to all PW at each contact in facilities and during outreach sessions at community level	ANC providers should distribute at least 30 IFA tablets, free to PW at each contact in health facilities; the number may vary based on the expected timing of the next ANC contact	ANC providers should distribute a 1–3‐month supply of IFA tablets, free to PW during facility ANC visits or at monthly group meetings	ANC providers should distribute a 1–2‐month supply of IFA and calcium tablets free to PW, at monthly outreach sessions, also at facilities. Tablets should be prescribed by the ANC provider and dispensed by the pharmacist. Community workers are permitted to distribute supplements to hard‐to‐reach women
Monitoring	ANC registers have specific column for IFA, content not specified	ANC registers have specific column for IFA, content not specified	ANC registers have specific column for IFA, content not specified	ANC registers have specific column for IFA, content not specified
*Weight gain monitoring*				
Protocol: weight gain	11 kg for normal BMI women,7 kg for overweight women and less than 7 kg for obese women	11.5–16 kg for normal BMI women, 12.5–18 kg for underweight, 7–11.5 kg for overweight and 5–9 kg for obese	10–14 kg, with an average of 12 kg	1.5–2 kg per month or 9–12 kg total in the last 2 trimesters of pregnancy. If gain is below 1 kg or above 3 kg in a month, refer to a doctor
Service delivery	‐Weigh at each ANC contact	‐Weigh at each ANC contact	‐Weigh at each ANC contact	‐Weigh at every ANC contact
	‐ANC providers should weigh PW in government, private and NGO health facilities; NGOs may weigh PW during home visits with the help of volunteers ‐Record weight in the ANC register	‐ANC providers should weigh PW in primary health care facilities ‐Weight should be recorded in the health facility register and in a Family Health Card.	‐ANC providers should weigh PW in hospitals, health centres and health posts ‐Record in the ANC register ‐Counsel women on amount of food based on weight gain	‐ANC providers should weigh PW in health facilities and during community outreach sessions for ANC ‐Weight should be recorded in ANC registers
Monitoring	ANC registers do not have specific columns for weight gain; for counselling on weight gain	ANC registers do not have specific columns for weight gain; for counselling on weight gain	ANC registers do not have specific columns for weight gain; for counselling on weight gain	ANC registers do not have specific columns for weight gain; for counselling on weight gain
*Dietary counselling*				
Protocol: dietary Counselling	‐Consume a variety of foods daily, green leafy and coloured vegetables and fruits, meat, fish, eggs, lentils, nuts, and grains ‐ Consume three main meals and two additional meals every day, add at least one fistful of rice and lentils in addition to the PW's pre‐ pregnancy meals	‐Messages should be linked to weight gain ‐In addition to usual diet of cereals/roots, consume green and orange vegetables, meat/fish, lentils/beans or nuts, milk products, green leafy and vitamin A rich vegetables ‐Add one extra meal	‐Consume foods from each of the six major food groups (fat sparingly, milk/yogurt/cheese, vegetables, meat, fruit, bread/cereals/other carbohydrates) ‐Add one extra meal during pregnancy	‐Consume foods from five specific food groups in addition to the staple cereal: pulses/lentils, milk/milk products, dark green leafy vegetables, vit A rich fruits and vegetables, citrus fruits. For non‐vegetarians: consume eggs/fish/meat ‐In the second and third trimesters, eat 3 meals and 2 snacks daily
Service delivery	‐ANC providers should counsel PW during contacts at community clinics on dietary diversity and number of meals ‐NGO community health workers should counsel PW during home visits, in group education sessions and hold food demonstrations	‐ANC providers should give messages during ANC visits at primary health centres ‐Community volunteers should give messages during home visits	‐ANC providers should counsel PW during hospital, health centre, and health post ANC visits and during group meetings ‐Community volunteers should give messages during home visits	‐ANC providers should counsel PW during outreach ANC sessions and give food demonstrations with the help of community workers ‐Community workers should counsel PW during home visits, community events and celebrations for pregnant women, and hold food demonstrations
Monitoring	No space to record in ANC registers	No space to record in ANC registers	No space to record in ANC registers	No space to record in ANC registers
*Counselling on breastfeeding*				
Protocol: breastfeeding counselling	ANC providers should remind PW about BF immediately after delivery	ANC providers should remind PW at each contact to initiate breastfeeding within 30 min to 1 h post delivery	ANC providers should remind PW about BF immediately after delivery	ANC providers should remind women about BF immediately after delivery, placing the newborn in skin to skin contact with the mother
Service delivery	‐ANC providers should remind PW in the third trimester during contacts at government, private and NGO facilities	‐ANC providers should give messages on early initiation of breastfeeding at each ANC contact in facilities	‐ANC providers should counsel PW at hospitals, health centres and health posts, in PW group education sessions	‐ANC providers should give messages during monthly outreach sessions
	‐Community health workers should counsel PW during home visits and in group sessions	‐Community volunteers should counsel PW during home visits	‐Health extension workers and community volunteers should counsel in home visits	‐Community workers should counsel PW during home visits and at group events and celebrations in communities
Monitoring	No space to record in ANC registers	No space to record in ANC registers	No space to record in ANC registers	No space to record in ANC registers

Abbreviations: ANC, antenatal care; BF, breastfeeding, BMI, body mass index, IFA, iron and folic acid; PW, pregnant women.

Source documents for Table 2 on national policies and service delivery practices.

Bangladesh: National Guideline on ANC DGHS/MOHFW, 2017; Maternal Health SoP MoHFW, 2017; NNS Operational Plan (April 2017); National Strategy on Prevention and Control of Micronutrient Deficiencies (2015–2024); Comprehensive Competency Training Module on Nutrition, NNS, IPHN; NNS Operational Plan (2017); National Neonatal Health Strategy and Guidelines (NNHS); DGHS Guidance Document for Continuity of Nutrition Services during COVID‐19 Pandemic (2020). Nguyen et al. (2015).

Burkina Faso: “Politique nationale multisectorielle de nutrition” and “Plan stratégique multisectorielle de nutrition” (March 2019); “Directives nationales sur les soins prénatals au Burkina Faso” (June 2019); Protocole de santé de la reproduction: Santé de la femme et du nouveau‐né de moins de sept jours (December 2018). Kim, Ouédraogo, et al. (2020).

Ethiopia: FMOH, Guidelines for the prevention and control of micronutrient deficiencies in Ethiopia, January 2016; National Guideline on Adolescent, Maternal Infant and Young Child Nutrition, April 2016; Management protocol on selected obstetric topics (ANC guideline) FMOH (2010); National Newborn and Child Survival Strategy Document, 2015/16‐2019/20; FMOH, July 2016; National Nutrition Program II, 2016–2020. Kim et al. (2020).

*India*: MOHFW, revised ANC guidelines (draft) 2020; National anaemia‐free initiative, 2018 (inclusive of revised dosage to 60 mg elemental iron); National guidelines on calcium in pregnancy, 2014; National Institute of Nutrition dietary guidelines 2010; MOHFW revised ANC guidelines (draft), 2020 MN chapter available on http://www.nceard.roshni-cwcsa.co.in; National Guidelines on Antenatal care for ANMs and Medical Officers, 2011; Facility‐based newborn care guidelines, 2013–2014; MOHFW National breastfeeding program, 2016. Nguyen et al. (2018).

#### 3.1.1 Micronutrient supplementation

Micronutrient supplementation guidelines vary across countries and do not consistently reflect WHO recommendations (Table 2). Government guidelines for IFA in India align with WHO's and provide specific doses (180 tablets during pregnancy, 60 mg elemental iron and 400 mcg folic acid daily) (Gov. of India, 2018), but the recommended dose for calcium supplementation is lower at 1 g (2 tablets daily of 500 mg each) (Gov. of India, 2014). Ethiopia recommends 90 IFA tablets during pregnancy (FMOH, 2016a). In Burkina Faso and Ethiopia, no recommendations were available for calcium supplements (FMOH, 2016a, MINISTERE DE LA SANTE, 2019). Multiple micronutrient supplements are not included in the national guidelines and protocols in Bangladesh (IPHN & MOHFW, 2015), Ethiopia and India. In Burkina Faso, multiple micronutrient supplementation is identified as an intervention in the Nutrition Multisectoral Strategic Plan but has not been adopted in routine ANC services.

All countries support IFA supplements being given at no cost to pregnant women through government services. IFA distribution in Burkina Faso and Ethiopia is permitted only by trained medical personnel located in facilities (FMOH, 2010; MoH, 2018a). India uses trained community workers to deliver IFA and calcium supplements to reach women and communities with low ANC attendance (MOHFW, 2011), and home visits in India and Bangladesh are permitted for delivering supplements (DGHS/MOHFW, 2017). Pharmacies and private clinics sell iron containing prenatal formulations in all countries. Burkina Faso, Ethiopia and India have flexible norms on the number of IFA tablets that can be provided at each ANC contact through government services, and the number is reportedly based on the timing of the pregnant woman's subsequent contact. In Bangladesh, 30 tablets is the number to be distributed at each contact. ANC registers in all countries have a space for recording IFA distribution but guidelines do not specify what to record and how to use the data. Neither global nor country recommendations specify the content of IFA counselling or key messages needed to facilitate adherence, such as, a total of 180 tablets should be consumed during pregnancy, risks of not adhering and how to manage side effects.

#### 3.1.2 Weight gain monitoring

Weighing women during ANC is included in all national guidelines but not monitoring of weight gain or counselling on weight gain. WHO (2016) references Institute of Medicine (2009) recommendations on maintaining healthy weight gain within a specified range that is tailored to pre‐pregnancy BMI categories. The lower end of the range aims to prevent undernutrition and foetal growth retardation, while the upper end aims to prevent excessive weight gain. Burkina Faso follows global recommendations for weight gain (MINISTERE DE LA SANTE, 2019), Bangladesh recommends less weight gain (DGHS/MOHFW, 2017), and Ethiopia and India do not base the recommended weight gain on a woman's pre‐pregnancy (or early pregnancy) BMI (DGHS/MOHFW, 2017; FMOH, 2016b). Details of calculating weight gain, counselling on weight gain, record keeping and use of weight or weight gain data are not specified either by WHO or by national protocols (Table 2).

#### 3.1.3 Dietary counselling

National guidelines in the four countries follow global recommendations (WHO, 2016) to counsel pregnant women on meeting their nutritional needs. Countries emphasise the importance of expanding the diversity of diets using a variety of locally available foods to reach the goal of five or more specific food groups consumed each day (FAO & FHI 360, 2016). However, guidance on developing locally relevant counselling to incorporate accessible and affordable nutrient rich foods or making seasonal adjustments are not present in global or national protocols. In Bangladesh and India, five nutrient rich food varieties in addition to the staple cereal and lentils/pulses are currently recommended (NIN & ICMR, 2020; IPHN & MOHFW, 2015). Guidelines in India advise women on food sources based on their vegetarian/nonvegetarian preferences. Adding meals and/or snacks is recommended by the countries to meet increased energy and macronutrient needs during pregnancy. Burkina Faso and Ethiopia suggest messages on additional meals when women are weighed (FMOH, 2010, MINISTERE DE LA SANTE, 2019). According to WHO (2016), countries with areas that are highly food insecure or those with little access to a variety of foods may consider distribution of balanced protein and energy supplements; this is not noted in national ANC policies and protocols. There is also no guidance globally or in national protocols for routine record keeping and using data on dietary counselling.

#### 3.1.4 Breastfeeding counselling

According to WHO (2018), health facilities that provide ANC should counsel pregnant women and their families about the benefits and management of breastfeeding (WHO, 2018). In addition to promoting exclusive breastfeeding during the first six months, counselling recommended for ANC services includes teaching women practical skills for initiating breastfeeding within the first hour after delivery and refraining from prelacteal feeds. ANC providers in the four countries are directed to remind pregnant women to initiate breastfeeding within the first hour after delivery and discuss the benefits of breastfeeding, but the guidance does not specify that pregnant women need to identify supportive birth attendants, obtain help from birth attendants to position the baby immediately after delivery for breastfeeding, and develop skills to initiate breastfeeding and manage common postpartum difficulties (FMOH, 2016b; MoH, 2018b; MOHFW, 2016). There is no guidance on routine record keeping and using data to improve breastfeeding counselling in ANC.

### 3.2 Coverage of ANC services and delivery of nutrition interventions

**Figure 1:**
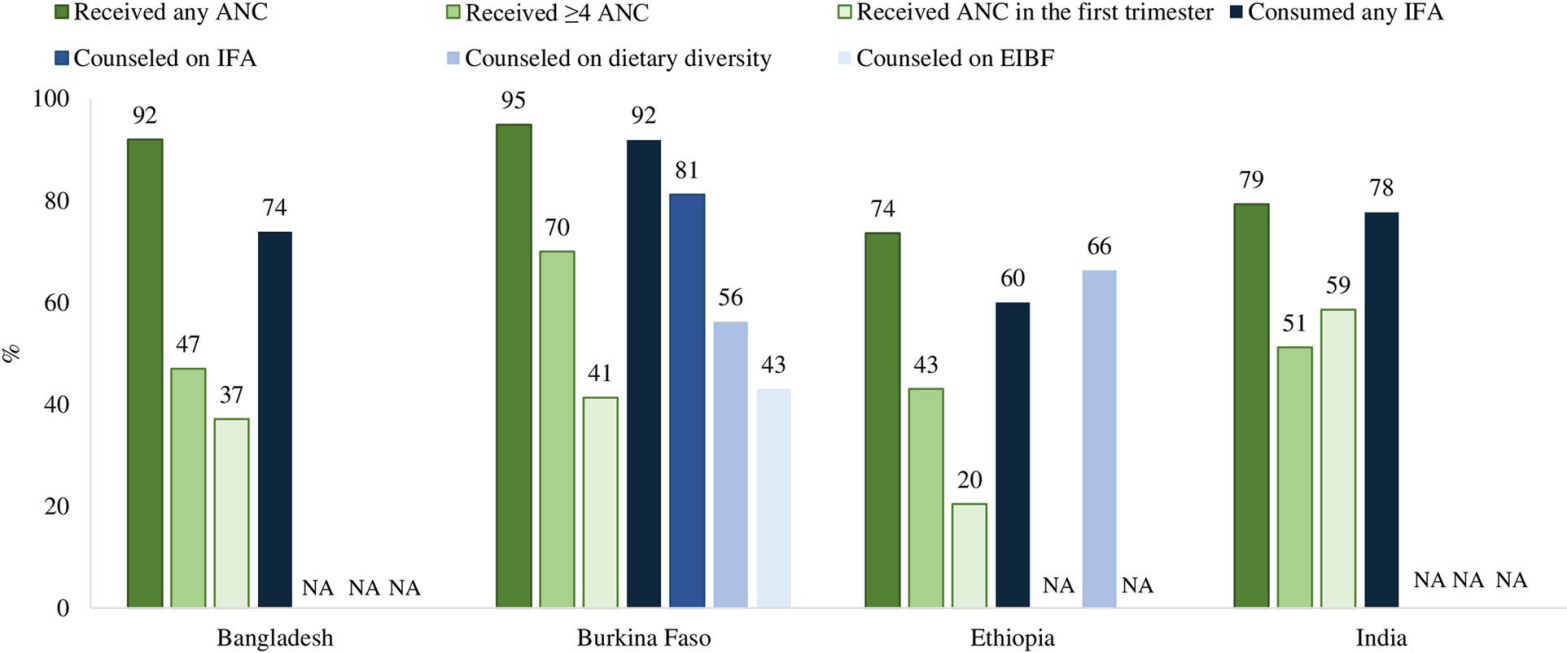
National coverage of nutrition interventions during ANC in Bangladesh, Burkina Faso, Ethiopia and India. *Source*: (1) Bangladesh DHS 2017–2018, Burkina Faso PMA2020 (2018) and DHS 2010, Ethiopia DHS 2016 and DHS 2019, India NFHS 2015‐16. (2) Data on “counselled on weight” were not available for any of the four countries. ANC, antenatal care; EIBF, early initiation of breastfeeding; IFA, iron and folic acid; NA, not available

**Figure 2:**
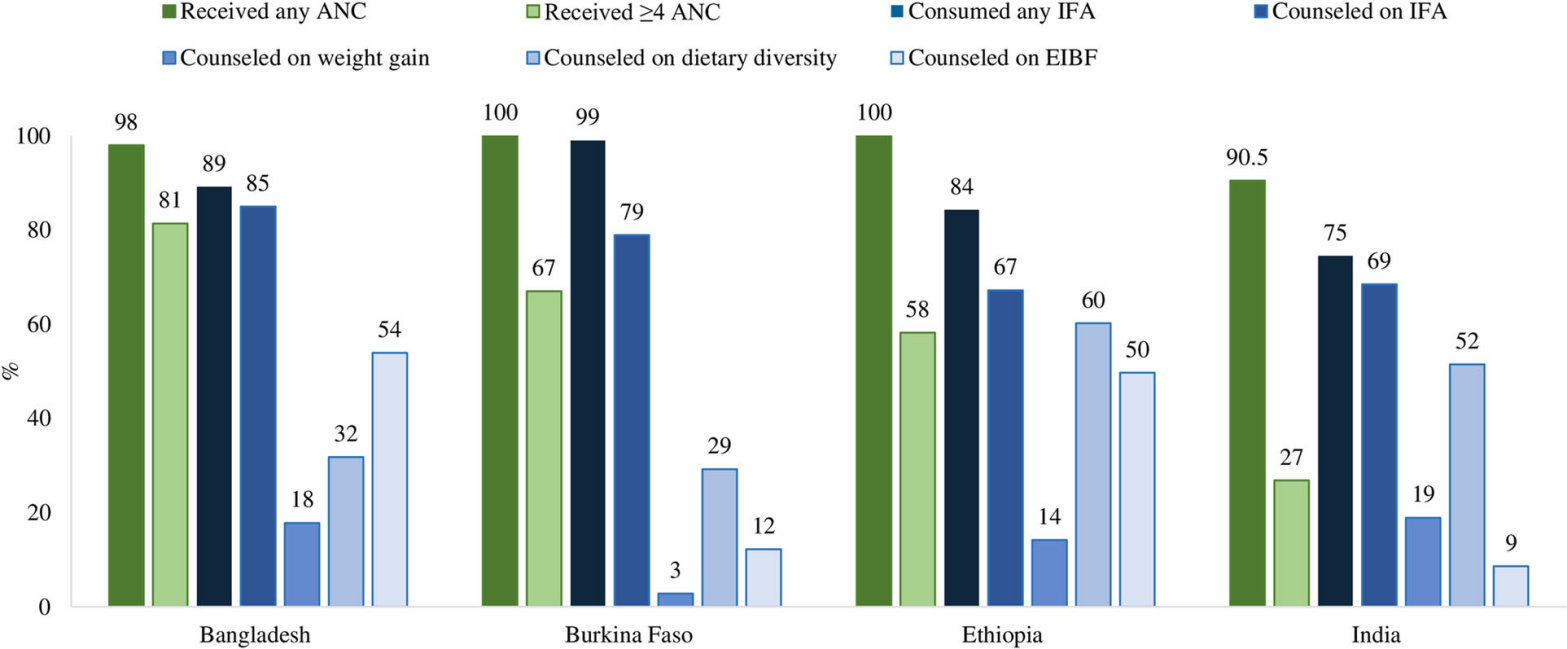
Subnational coverage of nutrition interventions during ANC in selected regions of Bangladesh, Burkina Faso, Ethiopia and India. *Source*: A&T/IFPRI Baseline Survey Reports. Bangladesh: Nguyen et al. (2015); Burkina Faso: Kim, Ouédraogo, et al. (2020); Ethiopia: Kim, Sununtnasuk, et al. (2020); India: Nguyen et al. (2018). Brief reports available at www.aliveandthrive.org. ANC, antenatal care; EIBF, early initiation of breastfeeding; IFA, iron and folic acid

At the national level, coverage of at least one ANC visit is high (from 74% in Ethiopia to 95% in Burkina Faso), however, the proportion of pregnant women in the four countries who sought ANC in the first trimester or completed at least 4 ANC visits was lower (Figure 1). Data is available for coverage of IFA supplementation, but not for coverage of counselling on IFA, weight gain, diet, and breastfeeding. Subnational data showed gaps between ANC coverage and nutrition service delivery, indicating missed opportunities for delivering nutrition interventions to pregnant women during ANC visits (Figure 2).

#### 3.2.1 Micronutrient supplementation

The consumption of any IFA tablets in national surveys varies from 60% in Ethiopia to 92% in Burkina Faso. A key component of micronutrient supplementation is counselling on adherence, but three out of four national surveys did not report coverage on this. Subnational surveys showed that coverage of counselling on IFA supplements varied by country, ranging from 67% in Ethiopia to 85% in Bangladesh. More women received ANC than those who received counselling on IFA, with the gaps ranging from 13 percentage points [pp] in Bangladesh to 33 pp in Ethiopia.

#### 3.2.2 Weight gain monitoring

According to subnational surveys, among all nutrition interventions, coverage of counselling on weight gain was lowest in all countries, ranging from 3% (Burkina Faso) to 18% (India). The proportion of women receiving any ANC was much higher than those receiving counselling on weight gain, indicating large missed opportunities. These gaps were 80 pp in Bangladesh, 97 pp in Burkina Faso, 86 pp in Ethiopia and 72 pp in India.

#### 3.2.3 Dietary counselling

Coverage of dietary counselling also varied by country according to subnational surveys, ranging from 21% in India to 60% in Ethiopia. More women received ANC than those who received counselling on dietary diversity; these missed opportunities ranged from 39 pp in India to 71 pp in Burkina Faso.

#### 3.2.4 Breastfeeding counselling

*A*ccording to subnational surveys, low proportions of pregnant women were counselled on early initiation of breastfeeding: 12% in Burkina Faso and 9% in India. These proportions are higher in Ethiopia (50%) and Bangladesh (54%), but still suboptimal.

### 3.3 Factors associated with delivery and utilisation of MNIs

Table 3 provides an overview of studies on factors associated with service delivery and utilisation of MNIs. Comprehensive and comparable studies were not widely available for the study countries and interventions. We found four studies directly related to the interventions from Ethiopia, three studies each from Bangladesh and Burkina Faso, and two studies from India (UP state). These studies used different designs including formative research, multivariable regression analyses, path analysis that explored health system factors, analyses on food access and demand, and assessments based on exit interviews with ANC clients.

**Table 3:** Overview of studies on factors associated with service delivery and utilisation of maternal nutrition interventions in ANC in Bangladesh, Burkina Faso, Ethiopia and India

Country, source, area	Study objective, design and sample	Interventions studied	Results: key factors associated with service delivery and uptake
*Bangladesh*			
Schuler (2015); Mymensingh, Dhaka, Rangpur districts	To design behaviour change interventions: in‐depth interviews, household trials and observations; 24 pregnant and seven recently delivered women. Key informant interviews; 8 husbands of pregnant women. Focus groups and in‐depth interviews with 55 women and their husbands in a study on gender norms in the same areas	IFA and calcium supplementation, dietary diversity, amounts of food	Access to free supplies of IFA and calcium was enabling, side effects were a concern for some Maternal diets were influenced by misperception that dietary diversity meant only costly foods, gender inequities and social norms (women expected to sacrifice their own wellbeing for the good of the family), husbands were willing and able to help, and family budgets were amenable to change with counselling (benefits for the child emphasised, specifying affordable foods)
Nguyen et al. (2017); Mymensingh, Rangpur, Kurigram, Lalmonirhat districts	To inform policies and programs: multivariable analysis of baseline survey; 600 pregnant and 2000 recently delivered women	IFA and calcium supplementation, dietary diversity	Number of ANC contacts, free supplies of IFA and calcium, maternal knowledge, family support were enabling
			Dietary diversity was influenced by maternal knowledge, beliefs, self‐efficacy, perceived social norms, and husband's support
Nguyen et al. (2018); Mymensingh, Rangpur, Kurigram, and Lalmonirhat districts	To inform policies and programs: path analysis was used to determine which of seven program implementation elements best explained maternal nutrition intervention results. Sample: total of 4000 recently delivered women, 437 health workers and volunteers interviewed in 2015 and 2016	IFA supplementation, weight gain monitoring, dietary diversity	Increased number of contacts and quality of counselling helped women to continue taking IFA and calcium supplements For dietary diversity, quality of counselling was key Results were associated with training quality, knowledge, reach of services, and counselling quality of health workers and volunteers
*Burkina Faso*			
Kim, Ouédraogo et al. (2020); Hauts Bassins and Boucle du Mouhoun regions	Baseline survey for evaluating the impact of interventions: 1920 recently delivered women	IFA supplementation, weight gain monitoring, dietary diversity and breastfeeding counselling	Delayed start of ANC, IFA side effects were barriers; support from family members (mostly husbands) to procure tablets and receive reminders to take IFA tablets, counselling by ANC providers and free supplies were positive factors
			Women were not counselled on weight gain and providers did not know the amount of recommended weight gain
			Barriers to dietary diversity included low maternal knowledge, perceived social norms and household food insecurity
			Maternal and provider knowledge of breastfeeding was not a factor in the low breastfeeding practices
Ky‐Zerbo et al. (2019); Hauts Bassins and Boucle du Mouhoun regions	To design behaviour change interventions: exit interviews; 120 pregnant women at ANC clinics. In‐depth interviews; 36 recently delivered women. Observations of group nutrition education; 11. In‐depth interviews; 24 ANC providers, 24 community health workers, 17 managers. Household trials; 48. Focus groups (18) and in‐depth interviews (48) with community influential persons	IFA supplementation, weight gain monitoring, dietary diversity, amounts of food	Inconsistent IFA supplies and poor quality of counselling on IFA were barriers Poor understanding and skills of ANC providers were barriers for weight gain monitoring; also needed better tools to calculate weight gain and to counsel Infrequent contacts and uncoordinated messaging were a barrier for dietary counselling; enabling factors included engaging families and husbands especially on dietary practices For early initiation of breastfeeding, preparing families of pregnant women and community elders in addition to pregnant women were enablers Lack of record keeping and use of data with follow up actions were barriers for IFA supplementation and counselling on dietary practice
			Integrating the interventions in routine district supervision were enabling
Kere (2020); Hauts Bassins and Boucle du Mouhoun regions	To assess maternal nutrition components of ANC in facilities: Exit interviews; 245 pregnant women after ANC visits and 42 women who delivered in the previous 72 h	IFA supplementation, weight gain monitoring, dietary diversity, breastfeeding counselling	Receiving adequate numbers of IFA tablets and being helped by family members to take a tablet daily were enabling Lack of weight gain counselling by ANC providers and low self‐efficacy of pregnant women in achieving recommended dietary diversity and weight gain levels were barriers Early initiation of breastfeeding was enabled by counselling during ANC Infrequent home visits by community agents to reinforce messages was a barrier
*Ethiopia*			
Kim, Sununtnasuk et al. (2020); SNNP and Somali regions	Baseline survey for evaluating the impact of interventions: 344 recently delivered women	IFA supplementation, weight gain monitoring, dietary diversity and breastfeeding counselling	Regional differences observed in IFA, potentially due to patterns of ANC contacts, inadequate supplies (in Somali), counselling and knowledge and family support (lower in Somali)
			Weight gain monitoring lacked counselling in both regions and weighing in ANC was less frequent in Somali
			Counselling on dietary diversity was low in Somali; dietary diversity perceived as costly
			Breastfeeding counselling during ANC was low in Somali
Hirvonen and Wolle (2019); Somali and SNNP regions	To inform policies and programs: secondary analyses of surveys: Feed the Future and PSNP evaluations, DHS, Price Survey, and Household Consumption‐Expenditure Surveys; identifies dietary diversity bottlenecks by regions	Dietary diversity of pregnant and nonpregnant women	Poor availability of nutritious foods in locally accessible markets was a barrier in Somali region but not in SNNP. In both regions, lack of knowledge and demand for the foods are barriers. The potential for increasing production and marketing of specific nutrient rich foods that are needed to fill maternal dietary gaps, was an enabling factor
Clemmons and Griffiths (2016); Amhara, Oromia, Tigray and SNNP	To design behaviour change interventions: formative research; 48 FGDs, 160 in‐depth interviews IDIs with pregnant and lactating women, husbands, elder women, agriculture and health extension workers, and community leaders	Dietary diversity of pregnant and lactating women	Social norms about not providing additional nutrition during pregnancy, and gender roles of being “self‐less” and not having access to household income and produce, are barriers for improving diets of pregnant women. Real or perceived financial constraints are barriers. Poor understanding of the problem is a barrier, for example, diet diversity is viewed as eating more varied dishes made from the usual cereal/tubers, not more food groups
Siekmans et al. (2018); Tigray, Amhara, Oromiya and SNNP Regions	To inform policies and programs: formative research; 32 FGD with pregnant women attending or not attending ANC; 8 FGD with influential community members; 56 in‐depth interviews with health providers, community workers, health staff	IFA supplementation	Late start of ANC and few ANC contacts are barriers, also inadequate IFA supply received even when they attend ANC, are barriers; women and ANC providers lack knowledge of the total number of IFA to be taken and why IFA is needed when PW are not ill. ANC providers not well trained to meet protocol targets, to record and counsel on IFA, and not given adequate supplies. Local supply chain bottlenecks (not national) are barriers
*India*			
Nguyen et al. (2019); Unnao, Kanpur Dehat districts in Uttar Pradesh state	To inform policies and programs: multivariable analysis of baseline survey; 600 pregnant, 1800 recently delivered women	IFA and calcium supplementation, weight gain monitoring, dietary diversity	Common enabling factors for taking IFA and calcium supplements, dietary diversity, and weight gain monitoring: women's knowledge, beliefs, and self‐efficacy; and family support and use of ANC services (frequent contacts and counselling) were significantly associated with practices
CMS (2015); Allahabad, Shahjahanpur, Siddharthnagar districts in UP state	To design behaviour change interventions: formative research; IDIs with 120 pregnant women, 37 mothers in law, 36 husbands, and household trials with 60 pregnant women	IFA and calcium supplementation, dietary diversity, amounts of food	Inadequate supplies of IFA and calcium, poor knowledge of benefits and IFA side‐effects were barriers
			Poor knowledge of dietary diversity and food amounts, and why specific foods are needed were barriers. Self‐efficacy among women was high for consuming green leafy vegetables, lentils, and milk. Yellow or orange plant foods were not considered normative; fish, meat, and milk products were costly, unavailable, and disliked. Lower prices and family support were enablers of dietary diversity

Abbreviations: ANC, antenatal care; CERTIS, Center for Studies and Research in Social‐health, Economic Technologies and Innovations; CMS, Center for Media Studies; DHS, Demographic Health Survey; FGD, focus group discussions; IDI, in‐depth interviews; IFA, iron folic acid; PSNP, Productive Safety Net Program; SNNP, Southern Nations Nationalities and Peoples' region.

#### 3.3.1 Micronutrient supplementation

In all countries, bottlenecks in the supply chain were common and affected ANC providers' ability to distribute sufficient tablets. The reasons for supply gaps included a lack of accurate forecasting, delayed requisitions, delayed reimbursement processes, poor knowledge of protocols and gaps in record keeping among service providers (Ghosh et al., 2020; Ky‐Zerbo et al., 2019; Zongo et al., 2019). Free provision and standardised specifications of prenatal IFA were positive factors for health services to manage service delivery for IFA.

Several factors influenced PW's adherence to the recommendations for micronutrients. Earlier start of ANC visits and higher number of visits, receiving counselling, maternal knowledge of micronutrient supplements and family support were significantly associated with higher IFA and calcium tablet consumption by pregnant women in multivariable analyses in Bangladesh and India (Nguyen et al., 2017, 2019). These enabling factors were also identified in formative research studies in Burkina Faso (Kere, 2020) and Ethiopia (Siekmans et al., 2018). Among barriers, bottlenecks in the supply chain affected women's ability to procure IFA and complete the protocol of 180 IFA tablets. Limited knowledge of pregnant women was found to be a common barrier including not knowing the importance of completing six months or 180 tablets during pregnancy, not understanding why healthy PW should consume the tablets in Ethiopia (Siekmans et al., 2018). Side effects and forgetfulness were barriers in Bangladesh (Schuler, 2015) and India (CMS, 2015).

#### 3.3.2 Weight gain monitoring

Information on factors associated with weight gain monitoring is limited. The key barriers for service delivery included lack of ANC provider's knowledge of adequate weight gain, and limited skills for calculating the amount of weight gained and counselling on weight gain. Multivariable analysis in India (UP state) showed that women's knowledge of adequate weight gain, family support, early start of ANC, completing four ANC visits, and receiving counselling on weight gain were associated with increased odds of having their weight monitored with greater frequency (Nguyen et al., 2019).

#### 3.3.3 Dietary counselling and dietary intake

Key factors associated with better dietary practices of pregnant women were maternal knowledge, confidence, self‐efficacy and beliefs (Nguyen et al., 2017). A high level of husband's support was enabling for women in Bangladesh, Burkina Faso and India, and associated with nearly two times greater likelihood of consuming diverse diets in India (UP state) and Bangladesh. Qualitative studies found gender inequities to be a barrier to consuming recommended diets in Bangladesh and Ethiopia, where social norms position women at lower priority to access food and consume food than men. Women were also not able to adhere to the recommendations because of seasonal or chronic lack of food in regions of Ethiopia (Hirvonen & Wolle, 2019), or they perceived the foods would be unaffordable in the case of Bangladesh and Ethiopia (Clemmons & Griffiths, 2016; Schuler, 2015). Formative work in all countries showed that women were not reached frequently enough during pregnancy, were not counselled adequately, and consequently, they lacked motivation and knowledge of why diverse foods were important and what affordable nutrient rich foods to consume.

#### 3.3.4 Breastfeeding counselling

Early start of ANC and frequent ANC visits by pregnant women were found to be associated with better breastfeeding practices. Mothers' perception that “milk doesn't form that early” led to lack of confidence in early initiation according to qualitative studies in Burkina Faso (Ky‐Zerbo et al., 2019). In addition to pregnant women, family members needed to be guided by ANC providers before the birth of the child in understanding why and how women should be supported to initiate on time. Obtaining family support can improve the effectiveness of counselling, when family members help to remind the PW and reinforce the practices recommended by service providers (Ghosh et al., 2020).

## 4 DISCUSSION

Although maternal nutrition is considered essential for achieving sustainable development goals (Victora et al., 2021) and ANC is the primary service platform for delivering MNIs, coverage of MNIs provided through ANC is low and many missed opportunities remain. Our paper examines why, where and when the gaps occur and highlights common strengths and weaknesses in the policies and programs related to MNIs delivered through ANC. Our findings also provide critical insights on barriers and enabling factors at individual, family, community and health system levels that are associated with either the delivery of nutrition interventions in ANC or related maternal nutrition practices followed by pregnant women or both.

Policies and protocols examined in our study at the global and national levels had several gaps, including the lack of specificity on: (1) counselling on adherence, record keeping and use of data for micronutrition supplementation; (2) adapting recommended weight gain ranges to local contexts and counselling on optimal weight gain; (3) determining contextualized dietary counselling content and quality; and (4) content and modality for breastfeeding counselling. Key MNIs were mentioned in national ANC guidelines. However, the content of guidelines and protocols were not consolidated and easily accessible, sometimes lacked specificity and did not consistently align with global recommendations. Nutrition interventions appeared to receive low priority among ANC services and there was lack of accountability for quality and coverage. ANC users in the countries experienced inadequate supplies of micronutrients and suboptimal counseling that was accompanied by limited community engagement, inconsistent record keeping and poor use of data by health staff.

For addressing national policy gaps, we suggest the need for closer coordination between nutrition and maternal health units within Ministries of Health, continued sharing of new evidence and review of relevant country data, developing operational specifications with insights gained from other country examples, and agreement on streamlined tasks and tools for ANC staff to feasibly incorporate key elements of nutrition interventions. Nutrition protocols that are embedded in ANC and nutrition policies, strategies and directives need to be more specific and consolidated in readily accessible documents. With suitable global and national guidance and technical assistance, achieving alignment in protocols by nutrition and ANC teams is possible. Joint assessments of data needs and developing standard operational practices for MNIs can be valuable starting points.

Our study indicates the need for greater clarity in global maternal nutrition guidelines to facilitate country‐specific adaptations. Examples include criteria and cut‐off points for adopting routine calcium and multiple micronutrient supplementation in ANC, dietary counseling and support for healthy weight gain in women with varying BMI and health conditions (e.g., diabetes, hypertension), and evidence‐based adherence strategies for micronutrient and dietary diversity protocols.

Our study identified substantial data gaps on coverage and quality of maternal nutrition services, which is consistent with previous findings in the Asia region (Torlesse et al., 2021). National surveys and routine monitoring data systems lacked process indicators needed for tracking nutrition interventions in ANC. Our findings align with global concerns on limited process monitoring indicators (Choufani et al., 2020; Gillespie et al., 2019; Heidkamp et al., 2021). For micronutrients, we found that the number of doses distributed per woman and counseling provided are not routinely recorded. Most women are weighed during ANC visits according to survey data and formative research, but weight gain and weight gain counseling are not recorded. Other indicators such as counseling on maternal diet, breastfeeding counseling and obtaining the support of family members, are also missing. The lack of data highlights an apparent lack of priority and accountability for maternal nutrition in health services. Efficient planning and targeting of scarce resources cannot be achieved without data. Previous evidence has shown that ongoing cycles of problem‐solving based on data and adjustments made in program delivery can improve worker performance (Bootwala, 2020). Accountability is essential to ensure that global and country actions are delivered with quality and equity to make a difference.

Our study highlights multiple barriers and enabling factors that influence the utilisation of MNIs; addressing these should be a priority of the health system. At scale programs face supply‐side health system level challenges such as micronutrient stock‐outs that can be anticipated and pre‐empted. Additional challenges at the system level include gaps in information systems and performance of vital tasks, such as counseling and community engagement for encouraging and facilitating utilisation of the key interventions. Demand‐related factors common across countries include late and infrequent care‐seeking for ANC, and poor adherence associated with gaps in knowledge, beliefs, self‐efficacy and family or community support. We found considerable variation in completion of at least four ANC visits, and this influenced the coverage and utilisation of nutrition interventions (Nguyen et al., 2017, 2019). Several nutrition interventions require sustained action at the household level, starting early in pregnancy and can be enabled or impeded by family members and social norms (Martin et al., 2020). Combining individual counseling with community‐based activities for involving husbands and other family members of pregnant women has shown impact on improving dietary diversity and household food security (Frongillo et al., 2019). Adherence to recommended maternal nutrition practices requires timely care‐seeking to obtain adequate supplies and reinforcement; repeated counseling throughout pregnancy to problem‐solve, build skills and confidence of PW; and active community‐level engagement for obtaining family support.

Our study is among the first to document current gaps in the content of a package of nutrition interventions within ANC protocols and existing health systems, thus providing critical information for filling missed opportunities in ANC. Using mixed methods including review of policies, programs and relevant literature, compiled data from national and subnational levels, and findings of formative research and stakeholder workshops, we offer rich evidence and suggest solutions to address bottlenecks in delivering nutrition interventions through ANC. We suggest how to begin to facilitate scale up of four proven interventions that provide substantial payoffs (WHO, 2016). Limitations of this study include the lack of nationally representative data on coverage and variations in sources of information.

## 5 CONCLUSION

Missed opportunities to address maternal malnutrition through health services remain widespread despite rising ANC coverage and evidence of negative impacts on maternal and child health outcomes. National policies acknowledge the need for implementing four priority nutrition interventions but do not provide specific guidance on or accountability for delivering them. Our review provides insights on where, when and why health system gaps are likely to occur, and key barriers and enabling factors associated with coverage and utilisation of MNIs. Attention is needed to strengthen micronutrient supply chains, establishment and use of information systems for accountability and performance improvement, enhance ANC provider knowledge and skills to deliver tailored counseling, and develop strategies for effectively engaging with families. ANC and nutrition departments within ministries of health need to fill policy gaps and develop country models for improving the provision and utilisation of nutrition interventions through ANC.

## CONFLICT OF INTERESTS

The authors declare that there are no conflict of interests.

## AUTHOR CONTRIBUTIONS

TS, PHN, MT, and SG conceptualised the review. The country policies and protocols were reviewed by SG, ZM, MZ, and TW. PHN, SK, and TS conducted the analyses and interpretation on service provision and utilisation. The manuscript was drafted by TS and PHN and edited by JE‐A and SK. All authors read and approved the final submitted manuscript.

## Supporting information

Supporting information.

## Data Availability

The data that supports the findings of this study are available in the tables/figures and in the supplementary material of this article.
