# Supplementary material for: Gaps in the implementation and uptake of maternal nutrition interventions in antenatal care services in Bangladesh, Burkina Faso, Ethiopia and India
Source: Matern Child Nutr. 2021 Nov 23;18(2):e13293. doi: 10.1111/mcn.13293 (PMC8932725; doi:10.1111/mcn.13293)
Supplement: Supplementary file 1 — Supporting information. [file MCN-18-e13293-s001.docx]

**Supplemental Table 1: Health and nutrition indicators in the four selected countries**

| Country | Year and survey | Infant Mortality Rate (Per 1,000 live births) | Maternal Mortality Ratio  Per 100,000 life birth (WHO *et al.*, 2019) | % Children stunted | % Pregnant women with 4+ ANC visits |
| --- | --- | --- | --- | --- | --- |
|  |  |  |  |  |  |
| Bangladesh | 2017-18 DHS | 38 | 173 | 30.8 | 47.0 |
|  | 2014 DHS | 38 | -- | 36.1 | 31.0 |
| Burkina Faso | 2014 MIS | NA | 320 | NA | 61.9 |
|  | 2010 DHS | 65 | -- | 34.6 | 33.7 |
|  | 2003 DHS | 81 | -- | 43.4 | NA |
| Ethiopia | 2019 DHS | 47 | 401 | 36.8 | 43.0 |
|  | 2016 DHS | 48 | -- | 38.4 | 31.8 |
| India | 2015-16 DHS | 41 | 145 | 38.4 | 51.2 |
|  | 2005-06 DHS | 57 | -- | 48.0 | 37.0 |

**References**

WHO, UNICEF, UNFPA, World Bank Group & United Nations Population Division 2019. Trends in Maternal Mortality: 2000 to 2017. Geneva, World Health Organization. <https://data.worldbank.org/indicator/SH.STA.MMRT>.
